# Cognitive reserve relates to executive functioning in the old–old

**DOI:** 10.1007/s40520-020-01758-y

**Published:** 2020-12-06

**Authors:** Joukje M. Oosterman, Michelle G. Jansen, Erik J. A. Scherder, Roy P. C. Kessels

**Affiliations:** 1 Donders Institute for Brain, Cognition and Behaviour, Radboud University, P.O. Box 9104, 6500 HE Nijmegen, The Netherlands; 2 Department of Clinical Neuropsychology, Vrije Universiteit Amsterdam, Amsterdam, The Netherlands; 3 Department of Medical Psychology and Radboudumc Alzheimer Center, Radboud University Medical Center, Nijmegen, The Netherlands; 4 Vincent Van Gogh Institute for Psychiatry, Venray, The Netherlands

**Keywords:** Cognitive reserve, Executive functions, Information processing speed, Episodic memory, Aging

## Abstract

Cognitive reserve (CR) is known to reduce or even protect against the negative effects of aging on cognitive functioning. Nonetheless, little is known about how CR influences the relationship between different cognitive abilities and age in the old–old. The goal of the present study was, therefore, to test the hypothesis whether, in the old–old, CR still modifies the relationship between age and cognitive functioning. Eighty-three adults (aged 71–94) without mild cognitive impairment or dementia residing in residential care facilities completed a detailed neuropsychological test battery. CR was estimated using a combination of educational attainment and an estimation of verbal intelligence. Moderation analyses revealed a significant effect for fluency and a trend for flexibility, showing that the negative relationship between age and cognitive performance is reduced as the level of CR increases. These results demonstrate that CR still influences the relationship between age and executive functions in adults of advanced age.

## Introduction

Cognitive reserve (CR) has become one of the most studied constructs in understanding the major variability in aging-related cognitive decline across individuals [1]. It has been hypothesized that CR increases the adaptability of cognitive or functional brain processes, thus acting as a buffer against the negative effects of aging-related brain pathology on cognition [1]. Although there are currently no established methods to capture CR directly, proxy measures of CR involve sociodemographic variables reflective of experiences that may promote CR (e.g., educational attainment and verbal intelligence) [1, 2]. Studies have indeed shown that higher levels of CR are associated with diminished cognitive decline in healthy aging [1, 2]. However, what remains less understood is the potential protective effect of CR at advanced ages (e.g., mean age over 80).

Previous studies that examined CR in the old–old have several limitations. First, although executive functions are considered the core functions underlying the compensatory mechanisms of CR [3], an intensive evaluation of diverse executive control processes in relation to CR in very old individuals is lacking. Most studies in the old–old examined CR in relation to global cognitive functioning or only a limited number of cognitive functions such as memory and processing speed [2, 4]. Second, studies that did examine multiple cognitive domains, including executive functions, mostly examined simple associations between CR and cognition [5, 6]. Therefore, it is unclear to what extent these findings may not merely represent main effects of, for instance, educational attainment [1]. Moreover, simple associations such as zero-order correlations cannot disentangle how CR attenuates the negative effects of aging and aging-related brain changes on cognition [1].

The present study aims to overcome these limitations and gain further insight into the effects of CR on cognition in the old–old. We examined how CR relates to cognitive test performance, primarily focusing on the different executive functions. Moreover, we examined how CR moderates the relationship between age and cognition. Due to the importance of executive functions in compensating for aging-related cognitive decline [3], we hypothesized that CR particularly influences the relationship between age and executive functions.

## Methods

### Participants

Eighty-three participants (M_age_ = 84.9, SD = 5.2, range 71–94; 29 men) were recruited in cooperation with four different homes for the elderly in Amsterdam, the Netherlands (see [7] for more details). We screened medical records of residents admitted to somatic wards for the following inclusion criteria: no neurological disease (e.g., dementia, Parkinson’s disease, stroke), no psychiatric disorder (e.g., schizophrenia, major depression), and no substance use disorder. To exclude individuals with potential cognitive impairments, the Mini Mental State Examination (MMSE) [8] was administered with a score of ≥ 24 as a requisite for participation (see Table 1 for descriptives).

**Table 1:** Characteristics of the study sample

Variable	*N*	Value
Age	83	84.9 (5.2)
Education level	83	
< Primary education	1	1.2%
Primary education	21	25.3%
Incomplete lower secondary education	13	15.7%
Lower secondary education	16	19.3%
Vocational education	19	22.9%
Higher secondary/professional education	7	8.4%
University degree	6	7.2%
NART IQ	80	98.7 (12.4)
MMSE	83	27.0 (1.7)
Memory		
RAVLT immediate recall	78	32.0 (9.4)
RAVLT delayed recall	78	5.7 (3.0)
Pattern recognition memory	76	18.9 (3.0)
Psychomotor speed		
TMT-A	81	98.4 (60.1)
Stroop Color-Word test W card	79	72.1 (19.3)
Stroop Color-Word test C card	79	59.6 (13.8)
Executive function		
Letter fluency	83	26.6 (10.9)
Category fluency	82	25.7 (9.5)
TMT-B	68	236.0 (125.6)
Stroop Color-Word test CW card	79	25.7 (12.3)
Digit Span total	83	10.8 (2.4)
SWM no. of between-search errors	75	66.1 (18.8)
IED no. stages completed	75	7.0 (2.4)
SOC no. of problems solved	68	6.2 (1.9)

All values represent means ± standard deviations, with the exception of education, which represents percentages. Stroop scores represent the number of correct responses within 45 s for a certain condition

*C* Color, *CW* Color Word, *IED* Intra-Extradimensional Set Shift, *MMSE* Mini Mental State Examination, *NART* National Adult Reading Test, *RAVLT* Rey Auditory Verbal Learning Task, *SOC* Stockings of Cambridge, *SWM* Spatial Working Memory, *TMT* Trail Making Test, *W* Word

### Neuropsychological assessment

A comprehensive battery of tests tapping memory, speed and attention, and executive functioning was administered, including the following tests [see 7, 8].

#### Episodic memory tests

The Rey Auditory Verbal Learning Test (RAVLT) was used as a measure of verbal episodic memory (immediate and delayed recall) and the Pattern Recognition Test (PRM; total correct) of the Cambridge Neuropsychological Test Automated Battery (CANTAB) as a measure of visual episodic memory.

#### Executive function tests

To fully grasp the complexity of executive functioning, we administered a wide array of tests to measure the following domains: flexibility, working memory, inhibition, fluency and planning. For flexibility, the Trail Making Test (TMT, using the TMT-B/TMT-A ratio score) and the Intra-Extradimensional Set Shift (IED, number of stages completed) task of the CANTAB were used. Working memory was assessed using the Digit Span (DS, forward and backward total correct) and CANTAB Spatial Working Memory (SWM, total between-search errors). Inhibition was measured with the 45 s version of the Stroop Colour-Word test. The interference score was used as primary outcome, which is based on the total correctly named words/colours on the Word (W), Colour (C) and Colour-Word (CW) cards, and calculated using the following formula: ${\text{interference}}\;{\text{ = CW }} - \;[({\text{W}} \times {\text{C}})/({\text{W}} + {\text{C}})]$. Fluency was assessed using category and letter fluency (for both total number correct productions). Finally, planning was assessed with CANTAB Stockings of Cambridge (SOC) (number of problems solved in minimal moves).

#### Information processing speed tests

We used the TMT part A (completion time) and the W and C cards (number of word/colours correctly named) of the Stroop test to measure processing speed.

### Cognitive reserve

Whereas previous studies mostly only used educational attainment to capture CR [2], we combined the level of educational attainment with verbal intelligence to obtain a more comprehensive and reliable proxy measure of CR [9]. For this, educational attainment was assessed with an ordinal rating scale based on the Dutch educational system that distinguishes between levels of education (rather than years of education): 1 = less than primary education, 2 = primary education, 3 = incomplete lower secondary education, 4 = lower secondary education, 5 = vocational education, 6 = higher secondary and professional education, 7 = university degree. The Dutch version of the National Adult Reading Test (NART) was used to estimate verbal intelligence.

### Analyses

All CR and neuropsychological test scores were *z* standardized. As we used two outcome measures of the RAVLT for episodic memory (immediate and delayed recall), *z* scores of immediate and delayed recall were first averaged into one score. A similar procedure was used for the two outcomes of the Stroop Colour-Word test for processing speed (C and W cards). Next, we used the z-standardized scores to calculate cognitive domain scores that covered flexibility (TMT-B/TMT-A, IED), working memory (DS, SWM), fluency (letter, category), information processing speed (TMT-A, Stroop Colour-Word) and episodic memory (RAVLT, PRM), as well as a CR score (education, NART). For inhibition and planning, the single test score was used. In case of missing data, we used the available scores to calculate the domain. If necessary, test scores were multiplied by − 1 such that a higher score indicates better performance.

Moderation analyses (5000 Bootstraps [10]) were performed to determine if CR moderates the relationship between age and the cognitive domain scores. The domain scores were used as dependent variable, age as main predictor, CR as moderator, and sex as covariate. We report both uncorrected and false discovery rate (FDR)-corrected *p* values to account for multiple comparisons. To estimate the effect sizes, we reported *R*^2^ and calculated Cohen’s *f*^2^ statistics using the following formula: Cohen’s $\, f^{2} = {{R^{2} } \mathord{\left/ {\vphantom {{R^{2} } {\left( {1 - R^{2} } \right)}}} \right. \kern-\nulldelimiterspace} {\left( {1 - R^{2} } \right)}}$. We interpreted 0.02 as a small effect size, 0.15 as a medium effect size and 0.35 as a large effect size.

## Results

Occasional missing data were present, most pronouncedly for the computerized CANTAB tests and the TMT-B, primarily due to the observed inability to complete or comprehend the (display of the computerized) test.

**Fig. 1:**
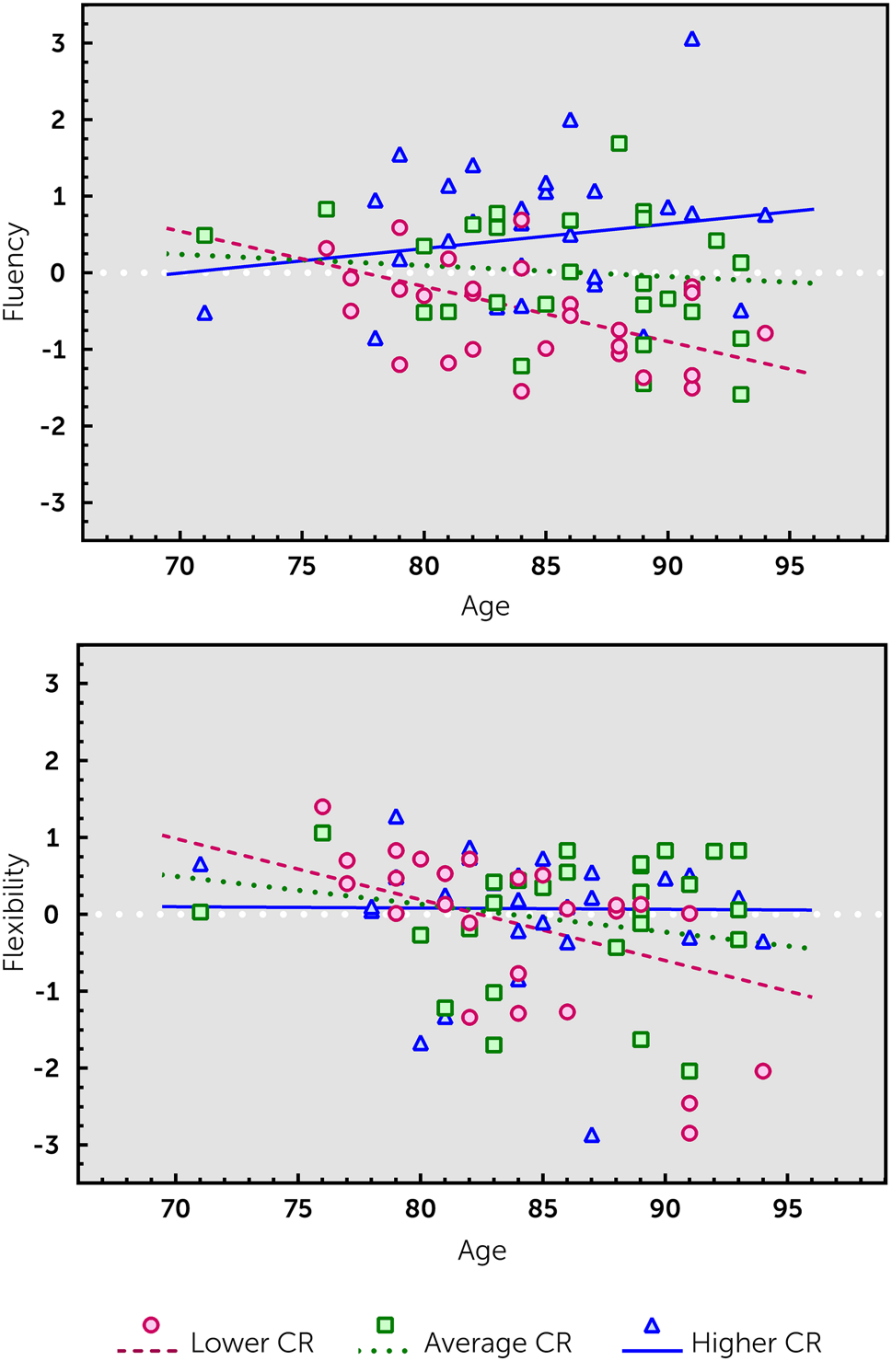
Moderating role of cognitive reserve (CR) on the relationship between age and fluency (upper panel) and age and flexibility (lower panel). The relationship is depicted for a lower reserve (circles/dashed line), average reserve (squares/dotted line) and a higher reserve (triangles/solid line). Note that the variables were centered for these analyses; to improve visualization of the effects, the interaction lines are superimposed on the raw scores of the age–cognitive domain relationship, distinguishing between three equal groups of CR (low, middle and high CR)

Results from the moderation analyses (see Table 2), after applying FDR corrections, showed main effects of CR for fluency, information processing speed, and working memory, indicating that a higher CR is associated with better cognitive performance. Furthermore, CR moderated the relationship of age with fluency performance (corrected *p* = 0.02, *R*^2^ change = 0.08), whereas a trend (uncorrected *p* = 0.06, *R*^2^ change = 0.04) was observed for flexibility. To further explore these effects, the relationship between age and these cognitive scores was plotted as a function of lower (− 1 SD), average (0 SD), and higher (+ 1 SD) CR (see Table 3 for the slope statistics). For both fluency and flexibility, a significant negative association with age was found only in individuals with a lower CR (all corrected *p* < 0.05), but not in individuals with an average or high CR (Fig. 1).

**Table 2:** Interactions between age and CR for different cognitive domains

Cognitive domain	Episodic memory	Working memory	Fluency	Flexibility	Planning	Inhibition	Information processing speed
Age	− 0.06 (− 0.09– − 0.02)****^†^	− 0.01 (− 0.04–0.02)	− 0.02 (− 0.05–0.02)	− 0.04 (− 0.07–0.00)*	− 0.05 (− 0.15–0.04)	− 0.31 (− 0.69–0.07)	− 0.04 (− 0.08– − 0.01)**
CR	0.09 (− 0.10–0.28)	0.32 (0.13–0.50)****^†^	0.55 (0.36–0.74)****^†^	0.15 (− 0.07–0.38)	0.14 (− 0.42–0.70)	0.38 (-1.87–2.63)	0.32 (0.12–0.52)***^†^
Sex	0.36 (0.01–0.71)**	0.12 (− 0.21–0.46)	0.15 (− 0.20–0.50)	0.41 (0.00–0.83)*	0.05 (− 0.98–1.08)	1.55 (− 2.62–5.71)	0.10 (− 0.27–0.47)
Age*CR	0.02 (− 0.02–0.05)	0.02 (-0.01–0.06)	0.05 (0.02–0.09)***^†^	0.04 (0.00–0.08)*	0.02 (− 0.08–0.12)	0.16 (− 0.25–0.56)	0.01 (− 0.03–0.05)
*F*_*interaction*_	*F*(1,76) = 1.06	*F*(1,78) = 1.93	*F*(1,78) = 8.86	*F*(1,75) = 3.55	*F*(1,63) = 0.14	*F*(1,73) = 0.58	*F*(1,76) = 0.24
∆*R*^2^_*interaction*_	0.01	0.02	0.08	0.04	0.00	0.01	0.00
*R*^2^_*total*_	0.22	0.15	0.32	0.16	0.02	0.06	0.19
Cohen’s *f*^2^_*total*_	0.28	0.17	0.47	0.19	0.03	0.06	0.23

*B* values are reported, together with lower and upper borders of the 95% confidence interval. Higher cognitive domain scores indicate better performance. Cohen’s *f*^2^ calculations are based on the unrounded *R*^2^ values

*CR* cognitive reserve (compound score of educational attainment and verbal intelligence estimate)

*Uncorrected *p* < 0.07

**Uncorrected *p* < 0.05

***Uncorrected *p* < 0.01

****Uncorrected *p* < 0.001

^†^Survived FDR corrections

**Table 3:** Slopes of the relationship between age and the cognitive domains as a function of CR

Cognitive domain	Fluency	Flexibility
Slopes		
Low CR	− 0.07 (− 0.11– − 0.02)*^†^	− 0.07 (− 0.13–-0.02)*^†^
Average CR	− 0.01 (− 0.05–0.02)	− 0.03 (− 0.07–0.00)
High CR	0.03 (− 0.01–0.07)	0.00 (− 0.05–0.05)

*B* values are reported, together with lower and upper borders of the 95% confidence interval. Slopes represent the relation between age and the cognitive domain score at a lower (− 1 SD), average (0 SD) and higher (+ 1 SD) level of CR

*CR* cognitive reserve (compound score of educational attainment and verbal intelligence estimate)

*p < 0.01

^†^Survived FDR corrections

The effect sizes (see Table 2) for the entire model ranged from small (planning, inhibition) to medium (episodic memory, working memory, flexibility, information processing speed) and large (fluency) effect sizes. A sensitivity analysis showed that, given a sample size of 83 with α = 0.05, the study was sufficiently powered (1 − β = 0.80) to detect a medium effect size (*f*^2^ = 0.16).

## Discussion

The goal of the present study was to examine whether CR influences the relationship between age and cognitive performance, with a particular focus on executive functioning, in the old–old. The results show that CR moderates the relationship of age with performance on fluency and, potentially, flexibility tasks. Corresponding to the notion that CR reflects one’s ability to alleviate the cognitive effects of aging-related brain pathology [1], these findings revealed that the negative correlation between age and cognitive performance levels is less pronounced as the level of CR increases. This study, therefore, clearly illustrates that even in a very old population, CR attenuates cognitive test performance. As cognitive, and particularly executive, functions play a profound role in functional independence in geriatric populations [11], these findings are of clinical relevance.

Our findings corroborate and extend previous studies showing that CR relates to cognitive functions in very old populations [2, 5, 6]. Our study provides new insights as we tested this for multiple executive functions, and analysed how CR modulates the relationship between age and cognition to focus on the hypothesized CR effects rather than the main effects of education [1]. Very little is known about how CR relates to the negative relationship between age and cognition in the old–old. From a theoretical point of view, one could hypothesize that CR may protect across the full life span, that is, as long as the aging-associated cognitive decline continues. Our study results corroborate this, by showing that even in very old individuals, CR still attenuates the negative age–cognition relationship for two executive function domains, although the moderation effect of CR on flexibility requires replication in larger studies.

The current study findings contrast those from a recent longitudinal study showing that education (as a proxy for CR) did not influence cognitive decline in these old populations [12]. As the current study relied on cross-sectional data, our findings may be confounded by cohort effects [13], although it has been reported that cross-sectional data may actually provide a very accurate indication of the true cognitive aging that occurs across the adult lifespan [13]. Moreover, previous work confirmed the interaction between CR and age, suggesting that the effects of CR actually increase with advancing age [14]. Likewise, the current study indeed revealed that with increasing age, the effect of CR becomes more pronounced (see Fig. 1).

The precise mechanisms via which CR exerts its protective role is still a topic of debate. According to one of the most prevailing theories, part of the protective role of CR may be exerted via increased capacity, efficiency or flexibility of existing neural networks [1]. From this perspective, CR may have a general effect on diverse cognitive functions that is supported by the already involved networks of brain regions. On the other hand, CR may work via compensatory mechanisms that are supported by alternative neural networks [1], mostly via increased involvement of prefrontal cortex processes and associated executive control processes [3]. However, a very recent study in unilateral frontal and non-frontal stroke patients failed to support this notion, showing that CR similarly influences cognitive performance in both patient groups [15]. The extent to which existing versus compensatory mechanisms are involved in the current study findings as well as in maintaining cognition in aging in general, requires further investigation.

To conclude, the current study suggests that CR attenuates the effects of age on executive functioning in old to very old adults. Considering the current worldwide double aging of the population, future studies are needed that examine the complex dynamics between age, neurodegenerative processes, and CR, as well as the functional and cognitive mechanisms in these old–old individuals that allow them to compensate for the effects of aging-related pathologies.

## Data Availability

The datasets generated during and/or analysed during the current study are available from the corresponding author on reasonable request.
